# Fear of Missing Out as a Predictor of Problematic Social Media Use and Phubbing Behavior among Flemish Adolescents

**DOI:** 10.3390/ijerph15102319

**Published:** 2018-10-22

**Authors:** Vittoria Franchina, Mariek Vanden Abeele, Antonius J. van Rooij, Gianluca Lo Coco, Lieven De Marez

**Affiliations:** 1 Department of Psychology and Educational Sciences, University of Palermo, 90133 Palermo, Italy; gianluca.lococo@unipa.it; 2 Tilburg School of Humanities and Digital Sciences, Tilburg University, 5037AB Tilburg, The Netherlands; 3 imec-mict-UGent, Department of Communication Sciences, Ghent University, 9000 Ghent, Belgium; trooij@trimbos.nl (A.J.v.R.); lieven.demarez@ugent.be (L.D.M.); 4 Department of Youth & Risky Behavior, Trimbos Institute, 3521 VS Utrecht, The Netherlands

**Keywords:** fear of missing out (FOMO), social media, problematic social media use (PSMU), phubbing, teenagers, adolescents, addiction

## Abstract

Fear-of-missing-out (FOMO) refers to feelings of anxiety that arise from the realization that you may be missing out on rewarding experiences that others are having. FOMO can be identified as an intra-personal trait that drives people to stay up to date of what other people are doing, among others on social media platforms. Drawing from the findings of a large-scale survey study among 2663 Flemish teenagers, this study explores the relationships between FOMO, social media use, problematic social media use (PSMU) and phubbing behavior. In line with our expectations, FOMO was a positive predictor of both how frequently teenagers use several social media platforms and of how many platforms they actively use. FOMO was a stronger predictor of the use of social media platforms that are more private (e.g., Facebook, Snapchat) than platforms that are more public in nature (e.g., Twitter, Youtube). FOMO predicted phubbing behavior both directly and indirectly via its relationship with PSMU. These findings support extant research that points towards FOMO as a factor explaining teenagers’ social media use.

## 1. Introduction

Behavioral addiction researchers argue that the psychological processes that explain problematic behavior require greater attention [1,2,3,4,5]. Understanding underlying processes is particularly relevant when examining problematic forms of (digital) media use. Digital devices, such as mobile phones, can be used and misused in a variety of different ways. It is likely that the way in which problem use manifests itself depends on the particular underlying psychological mechanism [6,7].

One psychological process that may underlie problematic digital media use is the Fear-Of-Missing-Out (FOMO). FOMO refers to the “pervasive apprehension that others might be having rewarding experiences from which one is absent” [8]. Persons who have a greater FOMO are assumed to have a greater desire to stay continually up-to-date of what others are doing, for example via the use of social media. According to Przybylski et al. [8], FOMO originates from psychological deficits in people’s competence and relatedness needs [9]. One way of satisfying these needs, the authors claim, is through the use of social media applications, because the dynamic nature of social media applications provides users with a consistent stream of social and informational rewards [10].

The purpose of the current study is to contribute to the extant body of research on FOMO in relation to social media use. The study has four aims. First, it investigates whether teenagers with higher levels of FOMO have more social media accounts (i.e., the breadth of social media use) and whether they access these accounts more frequently (i.e., the depth of social media use [11]) than teenagers with lower levels of FOMO. Second, assuming that teenagers with higher levels of FOMO are mostly motivated to check up on people in their personal social networks, we examine whether FOMO is a stronger predictor of the use of platforms that connect teenagers to their offline networks (e.g., Facebook, Snapchat) than of the use of platforms that connect to a largely unknown audience (e.g., Youtube, Twitter). Third, the study examines if teenagers with greater FOMO report higher levels of problematic social media use (PSMU) and, four, are more likely to report one particular form of problematic social media use, which is the use of social media during conversations with co-present others (cf. “phubbing”). These research aims are addressed with data from a large-scale cross-sectional survey that was administered to 2663 Flemish adolescents.

## 2. Theoretical Framework

People have always had a tendency to think about what others are thinking and doing [12]. In the 1970 and 1980 scholars already identified that some people developed anxieties around missing out, contemplating on the rewarding experiences that others might be having (e.g., in the context of romantic relationships) [13]. FOMO is thus not an entirely new concept.

FOMO can be understood as such an anxiety around missing out on rewarding experiences that results from people’s desire for interpersonal attachments [12]. This desire, which is grounded in people’s need to belong, is an innate and fundamental motivation which humans have [14]. People gratify this need by seeking belongingness to social groups. Social groups nowadays exist in both physical and virtual shapes and people have access to their social groups in both ways, online and offline. Social media, which can be defined as “Internet-based applications that build on the ideological and technological foundations of Web 2.0, and that allow the creation and exchange of User Generated Content” [15,16], offer a place where users can keep in touch with their social circles. Social network sites (SNS) such as Facebook or Instagram, for example, offer users an online connection to persons in their personal networks, facilitating the practice of keeping in touch.

Nowadays the digital world is considered an extension of the Self [17,18]. In addition to the personal mind and physical body, the Self can be thought to include people, places, physical possessions, as well as affiliation groups to which a person feels attached [19]. Social media platforms are a part of this: they are the digital portals to affiliation groups. In contemporary society we thus manage the affiliation network both offline and online; virtual groups are as real and important as the physical ones. Not being able to connect with those affiliation groups on social media may cause feelings of being out of touch with “real” life [17]. After all, losing or missing a person one is attached to, can bring feelings of loss and grieving as much as if a part of the Self was damaged [19].

The fear of being socially excluded plays a role in experiencing a FOMO [12]. Social exclusion produces a loss of belongingness, and therefore causes anxiety. Thus, when people cannot access their social media accounts, they might feel anxiety because of a fear that they are being socially excluded [12].

Social exclusion also elicits feelings of worthlessness [12]. These feelings lead people to the act of comparing themselves to others on social media [20] with the purpose of deciding upon their own personal value [21]. Social networks offer a place where consumers, particularly young generations, can continuously keep up with what peers are doing and checking on what they are missing out on (e.g., social events, life experiences, life opportunities, and so on and so forth). FOMO can thus drive social media use, as checking up on other people may lead to a temporary relief of one’s anxiety.

### 2.1. FOMO and the Use of Different Social Media Platforms

Users may use different media to gratify different needs [22,23,24]. This can explain differences in the popularity of certain social media platforms. In January 2015, the Global Web Index summary showed that the most popular social media platform was the social network site Facebook, immediately followed by YouTube, Twitter and Instagram. The same year Instagram’s popularity outperformed Twitter. According to some, this is because pictures are more effective than words in achieving self-presentational objectives, which are central motivations for social media use [25]. Given that each social media platform is characterized by its own features and affordances, it is relevant to differentiate between social media platforms in research on social media use: Different platforms may connect users to different persons and networks, and give access to different forms of information of which users may wish to stay up-to-date.

Current research findings reveal that FOMO is a predictor of the use of SNS with which users connect to people in their personal networks, such as Instagram [25] and Facebook [26]. Instagram use, for example, is found to be motivated by the desire to keep in touch with others [25] and to “to keep up with or gain knowledge about what others (i.e., friends, family and strangers) are doing” [27]. FOMO has also been found to predict Facebook use [28,29] and Instagram use [30,31]. The above study findings thus indicate that FOMO predicts the use of at least these social network sites, but potentially also the use of other social media platforms.

With respect to social media use, a difference can be made between the ”depth” and the “breadth” of one’s use, where the depth of use refers to aspects such as the frequency and duration of social media use, and the breadth of use refers to the variety of social media platforms that are actively used. For teens in particular, not only frequent use, but also the use of a broad variety of social media platforms may serve the purpose of relieving anxieties with regard to not knowing what others are thinking and doing, as the differences in the relational affordances of different platforms [32] mean that they may give access to at least partially different networks and contents. Hence, for the current study we expect that not only the depth, operationalized as the frequency of social media use, but also the breadth, operationalized as the number of active social media platforms teenagers use, are predicted by FOMO:

**Hypothesis** **1.** *Teens who experience greater FOMO, use social media more frequently (Hypothesis 1a;* i.e.*, the depth of social media use), and use more different social media accounts (Hypothesis 1b;* i.e.*, the breadth of social media use).*

### 2.2. FOMO and the Use of More Private versus More Public Social Media Platforms

As mentioned above, the use of different social media platforms may gratify different underlying needs. For example, a recent study shows that users significantly differ in the gratifications they derive from using Facebook, Instagram, Twitter and Snapchat [33]. One affordance in which platforms may differ is in the extent to which content is restricted to a (sub-)set of contacts, or fully public—in other words, whether content is shared with a mostly known versus a mostly unknown audience. On platforms such as Facebook, Instagram or Snapchat, people’s online social network usually overlaps with their offline affiliation group (e.g., Facebook/Snapchat contacts need to know each other’s names or phone numbers to see each other’s posts and profiles). Platforms such as Twitter or Youtube, on the other hand, usually make content accessible to a wider audience of mainly unknown individuals, and resemble a broadcasting platform rather than a platform in which content is restricted to people who have been accepted as “contacts”, “followers” or “friends”.

Given this difference in the public accessibility of platform content, it seems logical to assume that SNSs such as Facebook or Snapchat are more apt at providing relief from FOMO than, for example, video sharing platforms such as Youtube or microblogging services such as Twitter because the former provide greater relief from anxieties surrounding what friends and family are doing.

Indeed, platforms such as Facebook or Instagram are more personal SNS that enable teens to limit content accessibility to the desired public (e.g., friends or friends-of-friends). As a result, these SNS may be especially attractive venues for teens with a high FOMO because it lets them know what people in their primary affiliative groups are thinking and doing. We explore this assumption by asking the following research question:

**Research** **Question.** Is FOMO a stronger predictor of the use of more private social media platforms (that connect mostly to offline networks) than more publicly accessible social media platforms (that connect mostly to online networks)?

### 2.3. FOMO and Problematic Social Media Use (PSMU)

When social media use is excessive, it can become problematic. Several studies have addressed problematic social media use (PSMU) [34,35,36]. There is an ongoing debate in the literature around the differences between problematic social media use and a possible social media behavioral addiction [4,37]. An in-depth discussion of this debate goes beyond the scope of this work. In the current study, however, we use the term problematic social media use, which we define as an unhealthy excessive form of social media use, characterized by a lack of control over the behavior, and continued behavior despite adverse life consequences. Our focus is on revealing the factors that predict to problematic social media use in a general population of teenagers (i.e., the aim is not to diagnose or identify pathological cases).

As mentioned before, one of the aims of this study is to explore if teenagers with a greater FOMO report a higher levels of PSMU. Previous studies suggest that this is the case [38,39,40,41,42,43], and suggest that those who experience FOMO may try to relieve their anxiety by checking up on other people on social media. Ironically, however, the more people check their social media accounts, however, the more they may find events they are missing out on. Using social media to reduce the anxiety may end up to be another source of FOMO. Therefore this vicious circle may reinforce itself, gradually turning social media use into problematic social media use. Hence we expect:

**Hypothesis** **2.** Teens who experience greater FOMO, report higher PSMU.

### 2.4. FOMO and Phubbing

A final study purpose is to focus on FOMO as a predictor of one particular form of problematic social media use, which is the use of social media during conversations with co-present others. This practice is termed “phubbing” (derived from phone + snubbing), which refers to “the act of snubbing someone in a social setting by concentrating on one’s phone instead of talking to the person directly” [44,45]. Experimental studies show that phubbing negatively impacts relational outcomes such as impression formation [46].

Recent findings show that people prefer to use smartphones when going online [47]. Smartphones allows us to be in contact with our affiliation groups on social media, everywhere we are. Therefore, we assume that if people experience anxiety, they may temporary try to reduce it by accessing their social media accounts on their smartphones [48]. It is likely that those high in FOMO, who use social media to address their anxiety, may overuse social media on their smartphones in such a way that it intersects with their offline social interactions, leading them to phub their offline interaction partners.

Hence, the last aim of this study is to explore whether teens with a greater FOMO report to engage in phubbing behavior more frequently, and whether the latter relationship is mediated by PSMU:

**Hypothesis** **3a.** Teens with a greater FOMO, are more likely to use social media during conversations with co-present others (cf. “phubbing”).

**Hypothesis** **3b.** PSMU mediates the former relationship.

## 3. Method

### 3.1. Sample and Procedure

In Flanders, a consortium of non-profit organizations collaborates bi-annually on a large-scale survey project that examines the state of affairs of digital media ownership and use of Flemish youths. Apart from a large set of recurring questions, every edition of the survey includes a number of questions on topics that are considered relevant at the time.

The data gathered for the current study were part of the 2016 research project [49]. An omnibus survey was administered to the high school pupils of 11 geographically dispersed high schools in Flanders, Belgium. Using the information made available by the ministry of education, quota sampling was used to select schools, and—within the schools—years and classrooms. This procedure resulted in a final sample that is representative for the population in terms of gender, age and school track (see Van Waeg, D’Hanens, Dooms & Naesens [49] for further details).

Within each school, a local collaborator (e.g., the school’s information and communications technology coordinator) organized the survey administration process, according to a set of instructions provided by the project leaders. The survey was administered online, but to avoid self-selection bias, the data collection took part during school hours, in the computer rooms of the schools. Unfortunately, no response rates were registered. However, the local collaborators stated that few pupils did not receive permission to participate. In total, the responses of 3291 pupils were gathered. The project leaders subjected these responses to a rigorous data cleaning procedure, leading to removal of 452 responses that were either substantially incomplete, either contained multiple invalid responses to validation screening items. This procedure resulted in a final sample of 2663 pupils (57.1% girls; *M*_age_ = 14.87, *SD* = 1.67). This final dataset was distributed by the consortium to the collaborating researchers for further analysis.

Informed consent was collected from both the participating teenagers and their parents. Given the large sample-size, an opt-out procedure was used for collecting consent from parents. The university’s institutional review board approved the study.

### 3.2. Measures

#### 3.2.1. Breadth of Social Media Platforms Used

Based on interviews with young persons, a list of 25 frequently used social media applications was constructed. We adhered to Kaplan and Haenlein’s [16] definition of social media, which includes all platforms in which users can generate content that is (semi-)publicly available to others. The latter definition excludes platforms that focus exclusively on instant messaging (e.g., Whatsapp, Facebook Messenger). The list of included platforms can be consulted in Table 1. The breadth of social media use was assessed by asking for each platform whether the teenager had an active account (1 = yes, 0 = no), and then summing the total number of active accounts per participant.

#### 3.2.2. Depth of Social Media Platforms Used

The depth of social media use was measured by assessing for each active platform how frequently it was used (1 = less than once per week, 5 = multiple times per day). Questions with respect to the usage frequency of a platform were only answered by participants who had an active account for the platform. As visible in Table 2, a substantial number of platforms was used by (very) few participants. We opted to only include those platforms who were used by at least 5% of the sample. The analyses for Hypothesis 1a, concerning FOMO as a predictor of platform usage frequency (see Table 3 and Table 4) are performed on the basis of the subsample of users of each platform. 

#### 3.2.3. Private Versus Public Accessibility of Social Media Platforms Used

With respect to the private versus public accessibility of platforms, we consider *Facebook* and *Snapchat* as social media platforms on which content is generally less publicly accessible (i.e., content is oftentimes shielded off to a public of “approved” contacts), and *Youtube* and *Twitter* as social media platforms on which content is generally publicly accessible (i.e., content is usually accessible to everyone who visits the platform).

#### 3.2.4. Fear of Missing Out (FOMO)

The omnibus format of the survey implied a constraint on the number of items we could use. We chose to select four items from Przybylski et al.’s [8] 10-item FOMO-scale, as this scale had been pre-validated by the authors. In Przybylski et al.’s study, the ten scale items loaded on one factor, and were internally consistent. In the absence of information on the factor loadings of the individual items in the original study, we chose to select a subset of four items that reflect the diversity of the original scale items well. Those items were: “It bothers me when I miss an opportunity to meet up with friends”, “I fear my friends have more rewarding experiences than me”, “When I go on vacation or summer camp, I continue to keep tabs on what my friends are doing”, and “It is important that I understand my friends “inside jokes””. The items were measured on a 5-point Likert-scale (1 = completely disagree; 5 = completely agree).

A well-known risk of using a diverse set items to construct a short scale, is that internal consistency may be jeopardized [50]. Indeed, although an exploratory factor analysis revealed that the four items loaded onto one factor, with all factor loadings above 0.55, the total variance explained by the factor (42%) was below the advised 60% threshold, and the overall Kaiser-Meier-Olkin measure (0.65) indicated mediocre sampling adequacy. A further examination of the scale’s reliability, confirmed that the internal consistency of the scale was weak (*α* = 0.56), and revealed that it could not be further improved via item selection, as the highest inter-item correlation was 0.36 (*p* < 0.001). Appendix A shows the inter-item correlation matrix. Means and standard deviations can be consulted in Table 1.

A solution to the issue of low internal consistency, is to perform analyses with individual scale items, rather than with a scale variable. For the current study, however, such procedure would imply an inflation of results—particularly when answering Hypothesis 1a, which reduces the comprehensibility of the study findings. The alternative is to continue with a sub-optimal measure, knowing that the main risk of using a scale-measure that suffers from a weak Cronbach alpha, is underestimation of the real relationship [51]. In the context of the current study, we opted for the latter solution for reasons of comprehensibility, and thus work with the scale variable to answer Hypothesis 1. The risk of an underestimation of the real relationship should be kept in mind, however, when interpreting the results. For Hypotheses 2, 3 and 4, we report both the results using the FOMO scale variable and the individual scale items.

#### 3.2.5. Problematic Social Media Use (PSMU)

Problematic social media use was assessed using an adapted version of the C-VAT instrument [52], which is a scale based on the CIUS-scale that was developed and validated by Meerkerk, Van den Eijnden et al. [53] and Meerkerk [54]. The adapted version addresses social media use rather than videogaming. The items were measured on a 5 point Likert-scale (*α* = 0.82). The scale items, their means and standard deviations, and their factor loadings can be consulted in Table 1.

#### 3.2.6. Phubbing Behavior

At the time of constructing the questionnaire, we were unaware of scales measuring phubbing behavior. Hence, we measured phubbing behavior with three self-constructed items: “How frequently do you use your mobile phone during a conversation in a bar or restaurant?”, “How frequently are you engaged with your phone during a conversation?”, and “How frequently do you check social media on your phone during a personal conversation?”. The items were measured on a 5-point Likert scale, ranging from 1 (never) to 5 (almost all the time; *α* = 0.77). The scale items, their means and standard deviations can be consulted in Table 1.

#### 3.2.7. Control variables

Gender (1 = boy, 2 = girl), age and school track (1 = vocational, 2 = semi-vocational, 3 = academic) were included as control variables in the linear regression analyses.

### 3.3. Analyses

Hypothesis 1a states that teens who have a greater FOMO use social media platforms more frequently. We used ordinal regression analysis in to test this hypothesis because the frequency of use-measures have ordinal response scales. In the analyses with the frequency of use of Snapchat, We Heart it, Pinterest and Tinder as the dependent variables, the assumption of proportional odds was violated; this occurs more frequently in large samples, because minor violations of the parallel lines test may already be statistically significant [55]. Nonetheless, we advise to interpret these results with caution.

Hypothesis 1b states that FOMO positively predicts the breadth of social media use. The “breadth of social media use” variable was operationalized by counting the number of active social media accounts that teens have (min = 0, max = 25). We used multiple linear regression analysis to test the hypothesis, after assessing that the standardized residuals of the variable were normally distributed (and thus that the assumption of normality was not violated: because measurements gathered in large samples typically have very small standard errors, it is advised to assess normality on the basis of the absolute values of skewness and kurtosis rather than on Z-scores of skewness and kurtoses. Advised critical values for skewness, respectively kurtosis in large samples are 2, respectively 7 [56]. Using these guidelines, the skewness (1.22) and kurtosis (5.95) values of the residuals indicated that the assumption of normality for regression analysis was sufficiently met). 

To explore our research question on the comparative strength of the correlations between FOMO and private platforms on the one hand, and between FOMO and public platforms on the other, we first calculated the correlations, and next compared the strength of these correlations using Steiger’s Z-test [57] in Lee and Preacher’s [58] web-based software. The Steiger Z-test operates on the basis of Pearson correlations between two dependent correlations with one variable in common. Because the correlations have to be drawn from the same sample, we first recoded the ‘frequency of platform use’ variables so that persons without an active account received the lowest usage score (rather than a missing value). This recoding procedure ensured that there were 2663 responses for each variable. Next, we calculated the Pearson correlation coefficients, which form the input for the Steiger’s Z-test. The reader may notice that the Pearson correlation test is a parametric test, whereas the “frequency of platform use” variables are ordinal. However, the variables met the standard guidelines for skewness and kurtosis in large samples [56], and the Pearson correlation coefficients and the Spearman rho correlation coefficients were highly similar (i.e., for only two out of ten correlations the difference between the Pearson and the Spearman correlation coefficient exceeded a value of 0.03).

In social science research, scale variables are oftentimes treated as interval variables, based on the idea that the sets of items that comprise each scale form an index that represents an underlying latent factor [59]. The required assumptions for parametric testing were met. Hence, to examine Hypotheses 2 and 3, we fitted a mediation model using model 4 of Hayes’ [60]. Process macro for SPSS with FOMO as the independent variable, PSMU as the mediator and phubbing behavior as the dependent variable. The indirect effect was estimated for 5000 bootstrap samples with a 95% bias-corrected confidence interval.

## 4. Results

### 4.1. Descriptives

Before addressing the hypotheses and research questions, we briefly report some descriptive statistics for the media use variables. The means and standard deviations for the items of the FOMO-scale, the PSMU-scale and the phubbing scale can be consulted in Table 1.

For 25 social media platforms, we asked the teenagers in our sample whether they had an active account, and if so, how frequently they use it. In terms of active account ownership, Facebook (89% of teens with an active account), Snapchat (73%), Instagram (63%) and Youtube (60%) are the most popular social media platforms. The teenagers in our sample had on average 4.35 (Median = 4, Mode = 4, Min = 0, Max = 25) active social media accounts. The standard deviation (SD = 2.29) reveals that there is substantial variability between teenagers. Most teens who have an active account on Facebook, Snapchat, Instagram and Youtube, reported using the platform more than once per day (see Table 2). There are other social media platforms that are frequently used, such as the location-sharing platform Swarm (M = 4.26, SD = 1.09), but these oftentimes have small user bases (e.g., only 19% of teens has an active Swarm account).

The teenagers in our sample on average had a fairly neutral FOMO score (M = 3.06, SD = 0.69). Using a multiple regression analysis, we explored whether FOMO was predicted by gender, age and school track. Our analysis showed that girls (*ß* = 0.08, *p* < 0.001) reported a higher FOMO, whereas age (*ß* = 0.03, *p* = 0.109) and school track (*ß* = −0.01, *p* = 0.800) were no significant predictors, *R²* = 0.01, *F*(3.2659) = 5.96, *p* < 0.001).

### 4.2. FOMO as a Predictor of the Depth and Breadth of Social Media Use

Hypotheses 1a and 1b concern FOMO as a predictor of the depth and breadth of social media use. We tested Hypothesis 1a using ordinal regression analysis, with gender, age, school track and FOMO as the predictor variables, and the frequency of use of each respective social media platform that was used by 5% of the sample or more as the dependent variable.

Table 3 and Table 4 show the results. After controlling for gender, age and school track, FOMO positively predicted the use of the four most popular social media platforms: Facebook, Snapchat, Instagram and Youtube, as well as the frequency of using foursquare, Tumblr and Vine. These findings lend partial support to our hypothesis. While FOMO appears to be a modest predictor of the most common social media platforms, as well as of platforms that are used more rarely, it was not a consistent predictor of social media platform use.

With respect to the breadth of social media use (Hypothesis 1b), a multiple linear regression analysis with gender, age, school track and FOMO as predictors, revealed that gender (*ß* = 0.08, *p* < 0.001), age (*ß* = 0.20, *p* < 0.001), school track (*ß* = −0.05, *p* = 0.006) and FOMO (*ß* = 0.14, *p* < 0.001; *R²* = 0.08, *F*(4.2658) = 57.27, *p* < 0.001) were significant, positive predictors of the number of active social media accounts that teenagers have (H1b supported).

### 4.3. FOMO in Relation to the Public Accessibility of Platforms

We posited that FOMO would be a stronger predictor of social media use when the social media platform examined is a more private platform than when it as a more public platform (RQ1), because more private platforms such as Facebook or Snapchat connect teenagers more strongly to their offline ties, which can be considered the dominant affiliative group on which they want to keep tabs. We calculated Steiner’s *Z* to statistically compare the correlations between FOMO and Facebook, respectively Snapchat use on the one hand, and between FOMO and YouTube, respectively, and Twitter on the other hand (see Table 5 and Table 6).

The findings show that the correlations between FOMO and Facebook (*r* = 0.16, *p* < 0.001), respectively Snapchat use (*r* = 0.17, *p* < 0.001), are significantly stronger than the correlations between FOMO and YouTube (*r* = 0.00, *p* = 0.921), respectively Twitter use (*r* = 0.06, *p* = 0.002), thus lending support to our research question.

### 4.4. FOMO as a Predictor of PSMU and Phubbing Behavior

**Figure 1:**
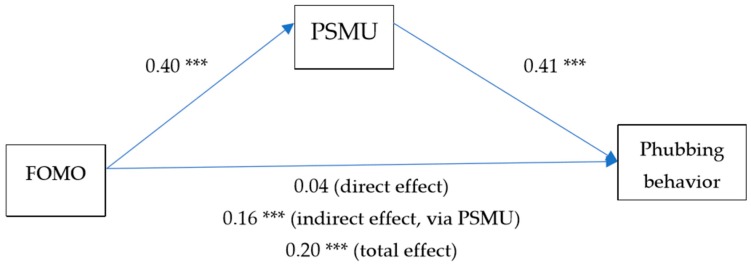
Mediation model of the relationship between FOMO, PSMU and phubbing behavior (*** *p* < 0.001). FOMO: Fear-of-missing-out; PSMU: problematic social media use.

Hypotheses 2 stated that FOMO positively predicts PSMU and Hypothesis 3a stated that FOMO positively predicts phubbing behavior. Hypothesis 3b stated that PSMU mediates the relationship between FOMO and phubbing behavior. We tested these hypotheses by estimating a mediation model. The results are depicted in Figure 1. All hypotheses were supported. FOMO has a direct, positive predictor of both PSMU (*ß* = 0.40, *p* < 0.001) and phubbing behavior (*ß* = 0.20, *p* < 0.001). When PSMU is accounted for, the relationship between FOMO and phubbing behavior weakens considerably. The indirect effect is significant (*ß* = 0.16, *p* < 0.001).

Given that the internal consistency of the FOMO scale was unsatisfactory, we performed the mediation analysis on the individual items of the FOMO scale (see Table 7). For three items, the mediation analysis resulted in a similar results pattern. For the item “It bothers me when I miss an opportunity to meet with friends”, however, the direct relationship with PSMU was much weaker (albeit still significant; *ß* = 0.05, *p* = 0.008), and the direct relationship with phubbing behavior was negative (*ß* = −0.04, *p* = 0.011).

## 5. Discussion

Drawing from the results of a survey study among 2663 Flemish teenagers, the aims of this study were fourfold: (1) to examine FOMO as a predictor of the depth and breadth of social media use; (2) to examine whether FOMO relates more strongly to the use of more private social media platforms than more public platforms; (3) to test whether FOMO predicts PSMU and (4) whether FOMO predicts, both directly and indirectly via PSMU, phubbing behavior.

With respect to the breadth of social media use (Hypothesis 1b), we found support for the hypothesis that teens who have a greater FOMO use a wider variety of social media platforms. With respect to the depth of social media use, the study findings partially support the hypothesis that teenagers with a higher FOMO use social media more frequently (Hypothesis 1a), as FOMO was identified as a predictor for the frequency of use of some, but not all social media platforms examined. We did find a consistent relationship with the usage frequency of the four most used platforms: Facebook, Snapchat, Instagram and YouTube. This finding suggests that the relationship between FOMO and the frequency of use of these popular platforms is generalizable to the population of at least Flemish teenagers. These findings also support the findings of earlier work that characterize FOMO as an intrapersonal characteristic that predicts the use of Facebook [28,29] and Instagram [30,31].

The relationships found between FOMO and the frequency of using less popular social media platforms such as Foursquare, Tumblr and Vine are more difficult to interpret, as these social media platforms differ considerably from each other in what they afford the user to do: Foursquare is a location-based social network site, Tumblr is a blogging service, and Vine let users share short videoclips. Future research may explore the relationship between FOMO and the use of these particular platforms more in-depth via the use of interviews with teenagers, as greater insight into their personal experiences with these platforms and their anxieties concerning missing out may shed new light on what makes these particular platforms attractive.

As mentioned above, a pertinent question is whether the social gratification provided by social media use sooths or aggravates the anxieties of teenagers; after all, studies on social comparison on social media platforms [61] suggest that exposure to other people’s social media accounts may make the experience of missing out on rewarding experiences even greater. A limitation of our study is its correlational nature, which prevents from making claims concerning the potential bi-directional causality of the relationship between FOMO and social media use. Future research may address this question via the use of longitudinal study designs that enable the modelling of cross-lagged path models.

It is essential for our understanding of FOMO to unravel how it resemblances, but also differs from other, associated personality factors. One factor that has been identified in the extant literature and that appears relevant to consider, is sociotropy [62], which refers to an insatiable need for belongingness to others, visible in an over-reliance on approval from others, which can go at the expense of personal autonomy. In recent work, sociotropy is linked to the ritualistic monitoring of, oftentimes multiple social media platforms [63]. Future research may explore how the fear of negative feedback or rejection that is typical for sociotropic individuals aligns with a fear of missing out.

We questioned whether FOMO would be a stronger predictor of more private social media platforms such as Facebook or Snapchat than of more public platforms such as YouTube and Twitter (RQ1). Our exploratory analysis suggests this is indeed the case. This is unsurprising, given that FOMO itself has been conceptualized and operationalized in the current study as a fear to miss out on what friends are thinking and doing, and information about these friends can be found mostly on platforms that connect to people who are part of one’s offline network. Nonetheless the stronger relationship between FOMO and these private platforms, we need to remark that FOMO still remains a weak, yet significant predictor of some more publicly accessible platforms (e.g., Tumblr). A pertinent question is what still drives those higher on FOMO to use the latter platforms, then. As mentioned above, qualitative research seems relevant to further expand on the affordances in which various social media platforms resemble or differ from one another, and how these affordances are perceived, valued and acted upon by those with a higher versus lower FOMO.

FOMO was found to predict PSMU (Hypothesis 2), a result that aligns with the findings from other recent studies which showed that greater FOMO is associated with more problematic internet and smartphone use [40,64,65]. Moreover, FOMO was also associated with phubbing behavior (Hypothesis 3a), consistently with previous research that showed a similar pattern of associations [44]. Interestingly, our results showed that the relationship between FOMO and phubbing behavior was mediated by PMSU (cf. Hypothesis 3b), accordingly to the proposed conceptual model by Chotpitayasunondh & Douglas [44], which found that FOMO was a positive predictor of smartphone addiction, and that smartphone addiction predicted smartphone behavior. Thus, adolescents who are high in their fear of missing out are more likely to overuse the social media and smartphones, which in turns leads them to phub their offline interaction partners [66]. Scholars in the field of problematic media use research advocate to invest greater effort into the study of the pathways that lead to problem behavior [6]. Our study findings represent such an effort, as they reveal that FOMO is an intra-personal characteristic that leads to phubbing behavior by inducing excessive, uncontrolled social media use.

The findings reported in this study are generalizable as they stem from a large-scale survey study that was administered to representative sample of Flemish teenagers. There are a number of limitations to the current study, however. We used a shortened, four item version of the FOMO-scale developed by Przybylski et al. [8] to assess teenagers’ FOMO. The internal consistency of the scale was unsatisfactory, which increases the risk of underestimating the real relationship (see Schmitt [51]). The low internal consistency for our scale illustrates the importance for research in this field to use complete and validated scales—for the reason of making reliable claims about one’s study, and for enabling valid comparisons between studies.

In light of the unsatisfactory internal consistency of our FOMO-scale, we tested Hypotheses 2 and 3 using both the scale variable and the individual items. This analysis revealed that for one item the relationship with phubbing behavior was reversed: The more teens agreed to feel bothered when missing an opportunity to meet with friends (i.e., indicative of a greater fear-of-missing-out), the less they report phubbing their interaction partner during a face-to-face interaction. This finding indicates that some teenagers attach great importance to face-to-face interactions with friends, leading them to prioritize these interactions over smartphone interactions. This finding suggests that it is relevant to further investigate how FOMO relates to relational behavior, not only online (in the form of social media use), but also offline.

Second, our study used a narrow definition of social media [16], which excludes mobile messaging applications. It is likely that those with a greater FOMO also heavily rely on the use of these messengers to soothe their anxieties about what others in their social networks are doing. The latter applications are different from social media, however, in that they are oftentimes used for small-group communication, and therefore are more dialogical in nature [67]. It is more difficult—if not impossible—to “lurk” in dyadic and small-group conversations, as they generally rely on a certain degree of reciprocity. With respect to FOMO, this difference in the interactional affordances of social media platforms and (mobile) instant messengers raises interesting questions. It may be the case that people with a high FOMO are particularly attracted to social media because they can lurk on these platforms without risking a label of “voyeur”, and without having to engage in reciprocal disclosures about themselves. As mentioned above, it seems relevant to explore the difference between FOMO and sociotropy [62] in this context, as active disclosures on social media platforms and messaging platforms may provide sociotropic individuals with a means to gather the social approval they long for, while those with a high FOMO may seek information about other people’s experiences without necessarily wanting to engage in interactions with them. Future research may explore this. 

In short, this study is valuable because it provides generalizable findings on the relationships between FOMO, social media use, PSMU and phubbing behavior among teenagers. As such, it can serve as a starting ground for future research. This research needs to look into the pathways via which FOMO leads to particular forms of (problematic) media use, and how these pathways are similar to or different from other pathways.

## 6. Conclusions

To conclude, and on the basis of the findings presented in this article, FOMO is an important factor explaining teenagers’ social media use. The present study found support for the hypothesis that teens who have a greater FOMO use a wider variety of social media platforms. Also, the present study found partially support for the hypothesis that teenagers with a higher FOMO use social media more frequently: FOMO was identified as a predictor for the frequency of use of some, but not all social media platforms examined. In particular, there was a consistent relationship between FOMO and the usage frequency of Facebook, Snapchat, Instagram and YouTube. Moreover, FOMO was found to predict PSMU. This result aligns with the findings from other recent studies which showed that greater FOMO is associated with more problematic internet and smartphone use [40,64,65]. Finally, FOMO was associated with phubbing behavior. Our results additionally showed that the relationship between FOMO and phubbing behavior was mediated by PSMU. Teens who are high in their fear of missing out are more likely to overuse the social media and smartphones, which in turns may lead them to phub their offline interaction partners [66].

## Figures and Tables

**Table 1:** Scale items, means and standard deviations.

**Fear-of-Missing-Out (FOMO)**			
**Items**		**M**	**SD**
1	I fear my friends have more rewarding experiences than me	2.33	1.11
2	It is important that I understand my friends’ “inside jokes”	3.09	1.05
3	It bothers me when I miss an opportunity to meet up with friends	4.16	0.90
4	When I go on summer camp or vacation, I continue to keep tabs on what my friends are doing	2.66	1.14
**Problematic Social Media Use (PSMU)**			
**Items**		**M**	**SD**
1	How frequently do you find it difficult to quit using social media?	2.89	1.19
2	How frequently do others (e.g., your parents or friends) tell you that you should spend less time on social media?	2.72	1.28
3	How frequently do you prefer using social media over spending time with others (e.g., with friends or family)?	1.89	0.97
4	How frequently do you feel restless, frustrated or irritated when you can’t access social media?	2.33	1.16
5	How frequently do you do your homework poorly because you prefer being on social media?	2.51	1.17
6	How frequently do you use social media because you feel unhappy?	2.31	1.23
7	How frequently do you lack sleep because you spent the night using social media?	2.44	1.35
**Phubbing**			
**Items**		**M**	**SD**
1	How frequently do you use your mobile phone during a conversation in a bar or restaurant?	2.39	0.99
2	How frequently are you engaged with your phone during a conversation?	2.13	0.96
3	How frequently do you check social media on your phone during a personal conversation?	1.89	0.92

**Table 2:** Percentage of teenagers with an account on various social media platforms and average frequency of use among account holders (1 = less than once/week, 5 = multiple times/day).

Social Media Platform	N	% of Total Sample	Average Usage Frequency		Less than Once per Week	Once per Week	Multiple Times per Week	Daily	Multiple Times per Day
			*M*	*SD*	%	%	%	%	%
Facebook	2360	89%	4.49	0.87	1.4	3.6	6.4	22.2	66.5
Snapchat	1937	73%	4.36	1.00	2.4	4.5	10.1	20.5	62.5
Instagram	1680	63%	4.36	0.97	1.5	4.4	15.7	31.8	46.6
YouTube	1596	60%	4.17	0.95	2.1	4.4	9.7	22.3	61.4
Google+	925	35%	2.67	1.40	28.2	21.5	19.8	16.4	14.1
Twitter	582	22%	3.63	1.35	8.8	14.9	19.4	18.6	38.3
Swarm	510	19%	4.26	1.09	3.7	5.5	10.8	20.6	59.4
We Heart It	329	12%	3.42	1.34	10.6	16.4	21.9	22.2	28.9
Pinterest	324	12%	2.6	1.34	28.1	21.6	24.4	14.5	11.4
Tumblr	298	11%	3.52	1.35	9.4	16.4	21.1	18.8	34.2
Vine	232	9%	3.12	1.38	15.9	19.8	23.3	18.5	22.4
Foursquare	172	6%	3.03	1.72	34.3	8.7	9.9	13.4	33.7
Tinder	120	5%	2.52	1.51	36.7	20	15.8	9.2	18.3
Kiwi	117	4%	3	1.58	25.6	18.8	13.7	13.7	28.2
Ask.fm	92	3%	3.6	1.60	20.7	6.5	12	14.1	46.7
Runkeeper	72	3%	2.17	1.10	31.9	34.7	23.6	4.2	5.6
Reddit	60	2%	3.07	1.48	20	20	18.3	16.7	25
Happening	48	2%	2.65	1.42	29.2	20.8	20.8	14.6	14.6
Vimeo	32	1%	2.91	1.61	34.4	6.3	15.6	21.9	21.9
Strava	25	1%	2.6	1.44	28	28	16	12	16
LinkedIn	24	1%	2.08	1.38	50	16.7	20.8	12.5	/
Periscope	15	1%	3.07	1.28	13.3	20	26.7	26.7	13.3
Endomondo	11	0%	2.73	1.74	36.4	18.2	9.1	9.1	27.3
Ello	8	0%	1.5	1.41	87.5	/	/	/	12.5
Meerkat	6	0%	2.33	2.07	66.7	/	/	/	33.3

**Table 3:** Gender, age, school track and Fear-of-Missing-Out (FOMO) as predictors of the frequency of Facebook, Snapchat, Instagram, Youtube, Google Plus and Twitter.

	Facebook			Snapchat			Instagram			Youtube			Google Plus			Twitter		
	PE	SE	Wald	PE	SE	Wald	PE	SE	Wald	PE	SE	Wald	PE	SE	Wald	PE	SE	Wald
Gender (boy)	−0.50	0.09	32.55 ***	−0.68	0.09	52.79 ***	−0.61	0.10	35.85 ***	0.72	0.10	56.21 ***	0.18	0.12	2.23	−0.25	0.15	2.58
Gender (girl)	a																	
Age	0.15	0.03	28.1 ***	0.02	0.03	0.30	0.12	0.03	14.36 ***	−0.01	0.03	0.14	−0.09	0.04	6.42 *	0.02	0.05	0.15
School track (voc)	0.41	0.14	8.72 **	0.40	0.14	8.00 **	−0.34	0.14	5.75 *	0.54	0.15	13.58 ***	0.51	0.17	8.79 **	0.36	0.25	2.18
School track (s-voc)	0.28	0.12	5.67 *	0.35	0.12	8.88 **	−0.01	0.12	0.00	−0.01	0.12	0.00	0.40	0.15	6.84 **	0.76	0.18	19.06 ***
School track (ac)	a																	
FOMO	0.48	0.07	55.09 ***	0.28	0.07	16.89 ***	0.34	0.07	22.48 ***	0.18	0.07	7.08 **	0.04	0.09	0.22	0.15	0.11	2.02
Pearson GOF	*X*^2^(1821) = 1635.34, *p* = 1.00			*X*^2^(1731) = 1842.31, *p* = 0.031			*X*^2^(1643) = 1716.37, *p* = 0.102			*X*^2^(1623) = 1621.94, *p* = 0.503			*X*^2^(1335) = 1370.00, *p* = 0.247			*X*^2^(1083) = 1131.58, *p* = 0.148		
−2LL GOF	*X^2^*(5) = 150.10, *p* < 0.001			*X^2^*(5) = 88.07, *p* < 0.001			*X^2^*(5) = 79.19, *p* < 0.001			*X^2^*(5) = 75.47, *p* < 0.001			*X^2^*(5) = 17.20, *p* = 0.004			*X^2^*(5) = 29.41, *p* < 0.001		
Nagelkerke R^2^	0.07			0.05			0.05			0.05			0.02			0.05		

* *p* < 0.05, ** *p* < 0.01, *** *p* < 0.001, Note 1: PE = parameter estimate, SE = standard error, Wald = Wald test statistic, voc = vocational, s-voc = semi-vocational, ac = academic, GOF = goodness-of-fit, a = reference category.

**Table 4:** Gender, age, school track and Fear-of-Missing-Out (FOMO) as predictors of the frequency of Swarm, We Heart It, Pinterest, Tumlbr, Vine, Foursquare and Tinder.

	Swarm			We Heart It			Pinterest			Tumblr			Vine			Foursquare			Tinder		
	PE	SE	Wald	PE	SE	Wald	PE	SE	Wald	PE	SE	Wald	PE	SE	Wald	PE	SE	Wald	PE	SE	Wald
Gender (boy)	−0.48	0.19	6.58 **	−1.41	0.58	5.87 *	−0.59	0.29	4.18 *	−0.41	0.29	2.01	0.19	0.24	0.64	−0.04	0.29	0.02	−0.11	0.34	0.11
Gender (girl)	a																				
Age	0.21	0.06	11.25 *	−0.12	0.07	2.69	−0.03	0.07	0.16	−0.03	0.07	0.16	−0.13	0.08	2.48	0.03	0.11	0.10	−0.17	0.11	2.36
School track (voc)	−0.58	0.25	5.34 *	0.65	0.31	4.36 *	−0.10	0.27	0.15	−0.50	0.33	2.30	−0.21	0.36	0.33	0.25	0.37	0.46	0.75	0.48	2.45
School track (s-voc)	0.36	0.22	2.78	0.01	0.24	0.00	−0.17	0.25	0.43	0.07	0.25	0.08	0.05	0.29	0.03	0.33	0.34	0.93	0.48	0.42	1.33
School track (ac)	a																				
FOMO	0.16	0.13	1.49	0.24	0.14	2.86	0.19	0.17	1.28	0.29	0.15	3.77 *	0.37	0.18	4.22*	0.47	0.21	5.26 *	0.04	0.23	0.03
Pearson GOF	*X^2^*(915) = 856.28, *p* = 0.917			*X^2^*(571) = 607.49, *p* = 0.141			*X^2^*(619) = 627.27, *p* = 0.400			*X^2^*(619) = 620.67, *p* = 0.474			*X^2^*(599) = 616.26, *p* = 0.304			*X^2^*(475) = 488.52, *p* = 0.324			*X^2^*(371) = 373.34, *p* = 0.456		
−2LL GOF	*X^2^*(5) = 28.53, *p* < 0.001			*X^2^*(5) = 12.53, *p* = 0.028			*X^2^*(5) = 6.24, *p* = 0.283			*X^2^*(5) = 689.76, *p* = 0.123			*X^2^*(5) = 8.29, *p* = 0.141			*X^2^*(5) = 7.47, *p* = 0.188			*X^2^*(5) = 4.26, *p* = 0.513		
Nagelkerke R2	0.06			0.04			0.02			0.03			0.04			0.05			0.04		

* *p* < 0.05, ** *p* < 0.01, *** *p* < 0.001, Note 1: PE = parameter estimate, SE = standard error, Wald = Wald test statistic, voc = vocational, s-voc = semi-vocational, ac = academic, GOF = goodness-of-fit, a = reference category. Note 2: The results for We Heart It, Pinterest and Tinder have to be interpreted with caution as the assumption of proportional odds was violated.

**Table 5:** Correlations between FOMO and frequency of use of less publicly accessible platforms versus more publicly accessible platforms.

Pearson’s *r*	Facebook	Snapchat	YouTube	Twitter
Snapchat	0.48 ***	1		
Youtube	0.13 ***	0.04 *	1	
Twitter	0.15 ***	0.19 ***	0.18 ***	1
FOMO	0.16 ***	0.17 ***	0.00	0.06 ***

* *p* < 0.05, *** *p* < 0.001, Note 1: For these analyses, participants with no active account on the platform were assigned a value of ‘0′ on usage frequency to ensure that the analyses were performed on the same sample size. Note 2: We used Pearson’s correlation coefficient, to enable the calculation of a Steiger Z value (see the subsequent set of analyses in Table 6). Note 3: The normality assumption was not violated.

**Table 6:** Comparison of Pearson correlation strength between FOMO and more private platforms versus FOMO and more public platforms.

Steiner’s *Z* (*r_FOMO, column var_* vs. *r_FOMO, row var_*)	Facebook	Snapchat	YouTube	Twitter
Snapchat	−0.41			
Youtube	6.37 ***	6.38 ***		
Twitter	4.18 ***	4.62 ***	−2.30 *	

* *p* < 0.05, *** *p* < 0.001.

**Table 7:** Mediation analysis results for the individual items of the FOMO scale and the FOMO scale variable.

	a	b	c	c’	Indirect Effect Estimate
FOMO (4-item scale)	0.40 ***	0.41 ***	0.04	0.20 ***	0.16 [0.14; 0.19]
Individual items					
I fear my friends have more rewarding experiences than me	0.21 ***	0.42 ***	0.00	0.09 ***	0.09 [0.07; 0.10]
It is important that I understand my friends’ “inside jokes”	0.15 ***	0.43 ***	−0.01	0.05 ***	0.06 [0.05; 0.08]
It bothers me when I miss an opportunity to meet up with friends	0.05 **	0.43***	−0.04 *	−0.02	0.02 [0.00; 0.04]
When I go on summer camp or vacation, I continue to keep tabs on what my friends are doing	0.22 ***	0.38 ***	0.09 ***	0.18***	0.09 [0.07; 0.10]

* *p* < 0.05, ** *p* < 0.01, *** *p* < 0.001. Notes: a = effect of FOMO on PSMU. b = effect of PSMU on phubbing. c = effect of FOMO on phubbing. c’= total effect of FOMO on phubbing.

## Appendix A

**Table A1:** Intercorrelation matrix of items measuring the Fear-of-Missing-Out (FOMO).

Items FOMO Scale	1	2	3	4
I fear my friends have more rewarding experiences than me	1	0.36 ***	0.17 ***	0.26 ***
It is important that I understand my friends’ “inside jokes”		1	0.25 ***	0.21 ***
It bothers me when I miss an opportunity to meet up with friends			1	0.15 ***
When I go on summer camp or vacation, I continue to keep tabs on what my friends are doing				1

*** *p* < 0.001.
